# Residential schools and the effects on Indigenous health and well-being in Canada—a scoping review

**DOI:** 10.1186/s40985-017-0055-6

**Published:** 2017-03-02

**Authors:** Piotr Wilk, Alana Maltby, Martin Cooke

**Affiliations:** 1 Department of Epidemiology & Biostatistics, Schulich School of Medicine and Dentistry, University of Western Ontario, London, Ontario Canada; 2 Department of Paediatrics, Schulich School of Medicine and Dentistry, University of Western Ontario, London, Ontario Canada; 3 Children’s Health Research Institute, London, Ontario Canada; 4 Department of Sociology and Legal Studies, University of Waterloo, Waterloo, Ontario Canada; 5 School of Public Health and Health Systems, University of Waterloo, Waterloo, Ontario Canada

**Keywords:** Residential schools, Indigenous health, Wellness, Colonialism, Historical trauma

## Abstract

**Background:**

The history of residential schools has been identified as having long lasting and intergenerational effects on the physical and mental well-being of Indigenous populations in Canada. Our objective was to identify the extent and range of research on residential school attendance on specific health outcomes and the populations affected.

**Methods:**

A scoping review of the empirical peer-reviewed literature was conducted, following the methodological framework of Arksey and O’Malley (2005). For this review, nine databases were used: Bibliography of Native North Americans, Canadian Health Research Collection, CINAHL, Google Scholar, Indigenous Studies Portal, PubMed, Scopus, Statistics Canada, and Web of Science. Citations that did not focus on health and residential school among a Canadian Indigenous population were excluded. Papers were coded using the following categories: Indigenous identity group, geography, age-sex, residential school attendance, and health status.

**Results:**

Sixty-one articles were selected for inclusion in the review. Most focused on the impacts of residential schooling among First Nations, but some included Métis and Inuit. Physical health outcomes linked to residential schooling included poorer general and self-rated health, increased rates of chronic and infectious diseases. Effects on mental and emotional well-being included mental distress, depression, addictive behaviours and substance mis-use, stress, and suicidal behaviours.

**Conclusion:**

The empirical literature can be seen as further documenting the negative health effects of residential schooling, both among former residential school attendees and subsequent generations. Future empirical research should focus on developing a clearer understanding of the aetiology of these effects, and particularly on identifying the characteristics that lead people and communities to be resilient to them.

## Background

The effects of colonization are apparent in all aspects of Indigenous peoples’ health and well-being [1], affecting not only their physical health, but the mental, emotional, and spiritual wellness [2]. It is well established that Indigenous peoples in Canada experience a disproportionate burden of ill health compared to the non-Indigenous population [3]. In large part, these health disparities have been a result of government policies to assimilate Indigenous peoples into the Euro-Canadian ways of life, leading to physical and emotional harms to children, lower educational attainment, loss of culture and language, and the disconnect of family structures [4–6]. Many of the illnesses and conditions that are disproportionately experienced by Indigenous peoples, including obesity, diabetes, and cardiovascular disease, have therefore been attributed to the lasting effects of colonialism, including the Indian Act, the reserve system, and residential schooling [7]. Loppie Reading and Wien [8] note that colonialism, a distal determinant of health, is the basis on which all other determinants (i.e. intermediate and proximal) are constructed.

Among colonial policies, residential schooling has stood out as especially damaging to Indigenous peoples. The residential school system was intended to eradicate the language, cultural traditions and spiritual beliefs of Indigenous children in order to assimilate them into the Canadian society [5, 6, 9, 10]. More than 150,000 First Nations, Métis, and Inuit children attended the church-run schools between their establishment in the 1870s and the closure of the last school in the mid-1990s [11]. As admitted by government and church officials, the explicit purpose of the residential school system was “to civilize and Christianize Aboriginal children” [10]. In addition to the cultural and social effects of being forcibly displaced, many children suffered physical, sexual, psychological, and/or spiritual abuse while attending the schools, which has had enduring effects including, health problems, substance abuse, mortality/suicide rates, criminal activity, and disintegration of families and communities [5]. Moreover, many of the residential schools were severely underfunded, providing poor nutrition and living conditions for children in their care, leading to illness and death [5].

These attempts of forced assimilation have failed, in part due to the resilience and resistance of many Indigenous communities [12]. Nonetheless, it is apparent that they have had profound effects “at every level of experience from individual identity and mental health, to the structure and integrity of families, communities, bands and nations” [6]. The concept of *historical trauma* suggests that the effects of these disruptive historical events are collective, affecting not only individual Survivors, but also their families and communities [13, 14]. According to Kirmayer, Gone, and Moses, historical trauma provides a way to conceptualize the transgenerational effects of residential schooling, whereby “traumatic events endured by communities negatively impact on individual lives in ways that result in future problems for their descendants” [14]. Recent findings suggest that the effects of the residential school system are indeed intergenerational, with children of attendees demonstrating poorer health status than children of non-attendees [9]. In fact, families in which multiple generations attend residential schools have been found to have greater distress than those in which only one generation attended [9]. Although this provides important evidence of the role of residential schooling in the current health and social conditions of Indigenous peoples, the links in the causal chain are not well understood, and there are many potential intermediate factors between residential school attendance and its effects on subsequent generations [14].

The consequences of residential schooling for Indigenous peoples in Canada have been known for some time, having been documented by the accounts of former attendees [15, 16]. These effects parallel experiences in the USA and Australia, where boarding or residential schools were also a key tool of assimilation [17]. In its final report, the Truth and Reconciliation Commission of Canada made 94 “calls to action” to redress the legacy of residential schools [18]. Among those related to health, the TRC admonished federal, provincial and territorial levels of government to acknowledge the effects of Canadian government policies (e.g. residential schools) and, working together with Indigenous peoples, to identify and close the gaps between Indigenous and non-Indigenous communities in health outcomes [18]. Although there have been some empirical studies of the effects of residential schooling on Indigenous peoples’ health, there has been no previous attempt to synthesize the evidence of these effects. The purpose of this scoping review is therefore to describe the current state of the literature regarding residential school attendance and the health and well-being of Indigenous people in Canada. In particular we ask; what are the health outcomes that have been empirically linked to residential schooling, what are the populations in which these effects have been identified, and whether effects are found among Survivors or also among other family members and subsequent generations. By summarizing the current literature and identifying needs for further research, this effort can contribute to our understanding of the effects of residential schooling on the health and wellness of Indigenous peoples.

## Methods

### Search strategies

The scoping review process for this paper was informed by Arksey and O’Malley’s methodological framework for scoping studies [19]. A scoping review is an approach used to map the existing literature on a particular general topic in order to understand the overall state of knowledge in an area [19]. Scoping studies therefore typically have broad research questions and focus on summarizing the available evidence [20]. According to Armstrong and colleagues, a scoping review also differs from a systematic review in that the inclusion/exclusion criteria can be developed in an iterative process, the quality of studies might not be discussed in the review, and that the synthesis tends to be more qualitative in nature with the review used to identify parameters and gaps in a body of literature rather than coming to a conclusion about the evidence for a specific effect or effects [21]. Although a scoping review may not describe research findings in detail, it provides a way of navigating the area of research where the range of material is uncertain [19]. Arksey and O’Malley suggest five stages in conducting a scoping review: (1) identifying the research question, (2) identifying relevant studies, (3) study selection, (4) charting the data, and (5) collating, summarizing and reporting the results [19]. These five stages were used to inform and guide the current literature review. The intent of this scoping review was to assess the extent and range of empirical research examining residential schooling and health outcomes among Indigenous peoples. This broad research question was established at the outset and was used to guide the subsequent stages of the review. In order to identify relevant literature, we conducted a search of nine electronic databases: Bibliography of Native North Americans, Canadian Health Research Collection, CINAHL, Google Scholar, Indigenous Studies Portal, PubMed, Scopus, Statistic Canada, and Web of Science. The search strategy and search terms were developed with the assistance of an academic librarian who specializes in First Nations studies. Broad search terms were used within these databases and are documented in Table 1.

**Table 1:** Search terms

(“residential school*”)	
AND	
(health OR wellness OR wellbeing OR “well-being” OR “well being” OR “Indigenous health”)	
AND	
(Aborigin* OR Indigenous OR “First Nation*” OR Métis OR Metis OR Inuit OR Native)	

The search results were downloaded into the reference management software Endnote (Endnote X7, Thomson Reuters, 2014), from which duplicates were removed. Inclusion was determined using the following criteria: (a) English-language source (or translated abstract), (b) analysis using primary or secondary data, (c) focus on an Indigenous population in Canada (e.g., First Nations, Inuit, Métis), and (d) focuses on residential school attendance and its relation to health. Grey literature addressing residential school attendance and health were also sought out to provide additional support, including government or organization reports, commentaries, or news bulletins.

**Fig. 1:**
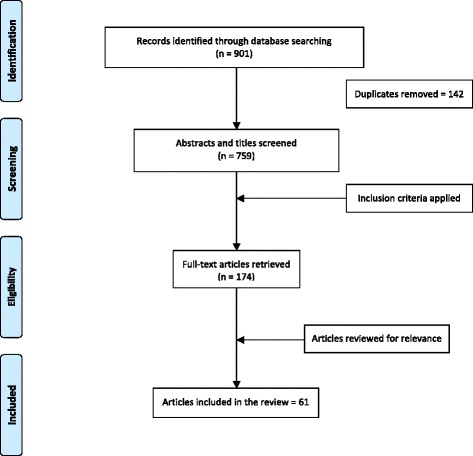
Scoping review search results

Selecting the articles for inclusion was completed in two steps. In the first stage, two reviewers screened titles and abstracts and citations that did not meet the inclusion criteria were removed. If the reviewers were unsure about the relevancy of an abstract, the full text of the article was retrieved and reviewed. At the second stage, the full texts of the articles were reviewed for final inclusion. The bibliographies of the full articles were hand-searched to identify further relevant references. Systematic or scoping reviews were not included in this scoping review; however, their reference lists were reviewed for pertinent references. A detailed chart depicting the search results is provided (Fig. 1). Following Arksey and O’Malley’s framework [19], a spreadsheet was created to chart the relevant data that is pertinent to the research question. The papers selected for inclusion were coded following similar categories used by Wilson and Young [22] and Young [23] in their reviews of Indigenous health research. The categories used includes: Indigenous identity group, geographic location, age-sex, residential school attendance, and health status. A description of each category is provided below. Data extraction was carried out by one of the researchers in an Excel database and was verified by another team member.

### Classification categories

Studies were classified according to the health outcomes examined, the Indigenous population affected, the geographic location of the study, and the age and sex/gender categories included in the study, and the type of residential schooling effect investigated.

#### Health outcomes

Although we distinguish specific types of health outcomes resulting from personal experiences and the intergenerational impacts of residential schooling, it is important to acknowledge that these outcomes do not occur independently, but exist in complex relationships with other effects [24]. The consequences of residential schools are wide-reaching and, according to Stout and Peters [24], may include, “medical and psychosomatic conditions, mental health issues and post traumatic stress disorder, cultural effects such as changes to spiritual practices, diminishment of languages and traditional knowledge, social effects such as violence, suicide, and effects on gender roles, childrearing, and family relationships”. Social, cultural, and spiritual effects of residential schools are often associated with physical, mental, and emotional health [24]. For the purposes of categorizing the types of outcomes described in the studies reviewed, it was necessary to impose somewhat arbitrary categories of physical health, mental health and emotional well-being, and general health, as described below.

1. Physical health: Health conditions may include arthritis, chronic back pain, rheumatism, osteoporosis, asthma, chronic bronchitis, emphysema, allergies, cataracts, glaucoma, blindness or serious vision problems that could not be corrected with glasses, epilepsy, cognitive or mental disability, heart disease, high blood pressure, effects of stroke (brain hemorrhage), thyroid problems, cancer, liver disease (excluding hepatitis), stomach or intestinal problems, HIV/AIDS, hepatitis, tuberculosis, or diabetes [25].
2. Mental health/emotional well-being: Mental health issues may include depression, anxiety, substance abuse (e.g. drugs or alcohol), paranoia, obsessive-compulsive disorder (OCD), panic disorder, post-traumatic stress disorder (PTSD), sexual dysfunction, personality disorders, stress, effects on interpersonal relationships, psychological or nervous disorders, and attention deficit disorder/attention disability. In addition, for the purposes of this review, suicide and suicide attempts or thoughts were also classified with mental health.
3. General health: A category related to general overall health was also included for papers that did not make references to a specific health outcome.

#### Indigenous identity group

Populations were also classified as either referring to a single Indigenous identity (First Nations, Métis, or Inuit) or a combination of identities (a combination of two single identity groups, or Indigenous and non-Indigenous identities).

#### Geographic location

For this review, we examined two aspects of geography. Firstly, we determined if the studies referred to Indigenous populations living on First Nations reserves,^1^ Northern communities, non-reserve rural areas, or in urban areas. Secondly, we identified the province or territory of focus in the paper.

#### Age-sex/gender categories

The health outcomes associated with residential school attendance might be different for men and women, or boys and girls. Studies were categorized by the age range and sex/gender of the participants.

#### Residential school attendance

Residential school attendance was classified as either personal attendance or familial attendance (i.e. parents, grandparents, aunts, uncles).

## Results

### Characteristics of the included studies

As depicted in Fig. 1, 61 studies were found that discussed residential schools in Canada and the health effects among Survivors, their families, or communities. The details of each study included in the review were provided in a chart and can be found in Table 2. The majority of papers were published in 2000 and later, with the exception of one published in 1999. Their sample sizes ranged from 1 to 51,080 and involved children, youth, and adults. Often, studies included men and women, various Indigenous identities, several geographic locations, and personal and familial residential school attendance.

**Table 2:** Summary of studies included in review

Author and publication year	Sample size	Indigenous identity group	Geographic location	Age-sex	Residential school attendance	Health status	Health related to residential school
T Anderson [39]	*N* = 2571	Inuit	NL, QC, NU, NT Off-reserve Northern	18+ years M/F	Personal Familial	Mental health/emotional well-being	Personal and familial residential school attendance only significantly related to men's mental distress
T Anderson and A Thompson [48]	*N* = 2925	Inuit	NL, QC, NU, NT Off-reserve Northern	15–54 years M/F	Personal Familial	General health	Personal and familial residential school attendance not significantly associated with self-reported excellent or very good health
SS Barton, HV Thommasen, B Tallio, W Zhang and AC Michalos [49]	*N* = 201	First Nations	BC Rural	M age = 63.5 (attended RS); M age = 61.2 (non-attendee) M = 93; F = 108	Personal	General health	Residential school attendees reported lower self-health scores compared to non-attendees
A Bombay, K Matheson and H Anisman [50]	*N* = 143	First Nations	ON, SK, BC, QC, AB, NB, MB, NS On/off-reserve Rural/urban	18–64 years M = 36; F = 107	Familial	Mental health/emotional well-being	Offspring of residential school Survivors appeared at increased risk for depression
A Bombay, K Matheson and H Anisman [51]	*N* = 399	First Nations, Inuit, Métis	ON Off-reserve	18–69 years M = 88, F = 311	Familial	Mental health/emotional well-being	Altered appraisals of threat were associated with higher levels of depressive symptoms relative to non-residential school adults
A Bombay, K Matheson and H Anisman [9]	N/A	First Nations	Canada-wide On-reserve	18+ years M/F	Familial	Mental health/emotional well-being	The more generations that attended residential school, the poorer the psychological well-being of the next generation
M Chongo, JG Lavoie, R Hoffman and M Shubair [52]	*N* = 24	Aboriginal	BC Urban	39–56 years M	Personal Familial	Mental health/emotional well-being	Many said historic trauma/residential school affected adherence to HAART, led to pain and/or abusing drugs, low self-esteem, self-blame, insecurity, fear, and resentment
MJ Cooke, P Wilk, KW Paul and S Gonneville [33]	*N* = 4060	Métis	Canada-wide Off-reserve Rural/urban/ Northern	6–14 years M = 2050, F = 2010	Parental Familial	Physical health	Residential school is a positive predictor of obesity among younger boys/girls but a negative predictor among older girls
RR Corrado and IM Cohen [5]	*N* = 127	First Nations	BC	17–81 years M = 89, F = 38	Personal	Mental health/emotional well-being Physical health	PTSD, substance abuse disorder, and major depression among residential school Survivors. Chronic headaches, heart problems, and arthritis also common
KJP Craib, PM Spittal, SH Patel, WM Christian, A Moniruzzaman, ME Pearce, L Demerais, C Sherlock, MT Schechter and P Cedar Project [37]	*N* = 512	Aboriginal	BC Urban Off-reserve	14–30 years M = 247, F = 265	Familial	Physical health	Having at least one parent who attended residential school was an independent risk factor for HCV infection
T DeBoer, J Distasio, CA Isaak, LE Roos, S-L Bolton, M Medved, LY Katz, P Goering, L Bruce and J Sareen [53]	*N* = 504	Aboriginal	AB Urban Off-reserve	Age N/A M = 320, F = 184	Personal Familial	Mental health/emotional well-being	Residential school history (particularly father’s history) and a number of mental and physical health conditions were significantly associated with volatile substance use
D Dionne [44]	*N* = 5	First Nations	AB On-reserve	47–71 years M = 1, F = 4	Personal Familial	Mental health/emotional well-being	Participant co-researchers explained addiction to drugs and alcohol as a coping mechanism
D Dionne and G Nixon [54]	*N* = 5	First Nations	AB On-reserve	47–71 years M = 1, F = 4	Personal Familial	Mental health/emotional well-being	First Nations people and their family members suffered from trauma, shame, marginalization, institutionalized conditioning and abuse
RF Dyck, C Karunanayake, B Janzen, J Lawson, VR Ramsden, DC Rennie, PJ Gardipy, L McCallum, S Abonyi, JA Dosman, et al. [32]	*N* = 874	First Nations	SK On-reserve	17–29 years M = 431, F = 443	Personal Familial	Physical health	Participants who attended residential school had slightly higher prevalence of diabetes than those that did not, but not statistically significant. Those with parent or grandparent residential school history also did not significantly predict diabetes
B Elias, J Mignone, M Hall, SP Hong, L Hart and J Sareen [41]	*N* = 2953	First Nations	MB On-reserve	18+ years M/F	Personal Familial	Mental health/emotional well-being	Attendees with abuse history likely to have history of suicide thoughts or attempts. Abuse history for non-attendees more likely for those with multi-generational residential school exposure
D Feir [29]	*N* = 4939	First Nations, Inuit, Métis	ON, MB, SK, AB, BC On/off-reserve Rural/urban	7–15 years M/F	Familial	General health	Children who had a mother that attended residential school fared better on numerous health dimensions than children whose mother did not attend
IM Findlay, J Garcea and JG Hansen [55]	*N* = 105	First Nations, Métis, non-status Aboriginal, other	SK Urban	18–64 years M = 32, F = 72, Other = 1	Personal Familial	General health	In part because residential school, as few as 6-11% reported physical, mental, emotional, and spiritual well-being as excellent
First Nations Regional Longitudinal Health Survey (RHS) [42]	*N* = 22,602	First Nations	Canada-wide (excl. NU) On-reserve	0–11 years (C) 12–17 years (Y) 18+ years (A) M/F	Personal	Mental health/emotional well-being Physical health	(C) No effects of familial residential school history (Y) Youth who had at least one parent attend residential school were more likely to have thought about suicide (A) Increased susceptibility to mental and physical health effects resulting from attendance at residential school
First Nations Information Governance Centre (FNIGC) [25]	*N* = 21,757	First Nations	Canada-wide On-reserve	0–11 years (C) 12–17 years (Y) 18+ years (A) M/F	Personal Familial	Physical health Mental health/emotional well-being General health	(C) Emotional or behavioural problems not associated with familial residential school history (Y) Intergenerational impacts of residential school related to depressive symptoms (A) Attendees more likely to be diagnosed with at least one chronic condition, smoking (maternal), and report poorer overall health and well-being
H Ghosh [30]	*N* = 20	First Nations	ON Off-reserve Urban	21–77 years M = 3, F = 17	Personal	Physical health	Consumption of a higher concentration of carbohydrates at residential school partly indicative of higher incidences of diabetes among First Nations people.
JP Gone [56]	*N* = 1	First Nations	MB On-reserve	50’s F	Personal	Mental health/emotional well-being	Traumatic stressors caused by residential school related to historical trauma. Enduring problems through adulthood, (e.g., alcoholism, religious alienation, and troubled relationships)
C Hackett, D Feeny and E Tompa [28]	*N* = 14,280	First Nations, Inuit, Métis	Canada-wide Off-reserve Rural/urban/ Northern	18+ years M, F	Familial	General health Mental health/emotional well-being	Familial residential school attendance was associated with lower self-perceived health and mental health and higher risk for distress, suicidal ideation and suicide attempt
GK Healey [35]	*N* = 20	Inuit	NU Northern	Parents of youth 13–19 years M = 3, F = 17	Personal Familial	Physical health	Parents discussed sexual health in the context of historical community events related to settlement and/or residential school
G Healey [34]	*N* = 20	Inuit	NU Northern	30–58 years M = 3, F = 17	Personal Familial	Physical health	Traumatic experiences of the settlement and residential school era impact present-day family relationships and parent-adolescent communication in general and specifically sexual health
HA Howard [31]	*N* = 124	Indigenous	Canada-wide	18–86 years M = 45, F = 79	Personal Familial	Physical health	Residential school contributed to the urbanization of Indigenous people and to their health problems, in this case to eating habits affecting diabetes
Y Iwasaki and JG Bartlett [57]	*N* = 26	First Nations, Métis	Western Canada Urban	26–69 years M = 9, F = 17	Personal	Mental health/emotional well-being	Some Indigenous individuals with diabetes described cumulative stress due to their traumatic experiences in residential schools
Y Iwasaki and J Bartlett [58]	*N* = 26	First Nations, Métis	Western Canada Urban	26–69 years M = 9, F = 17	Personal	Mental health/emotional well-being	Some Indigenous individuals with diabetes described cumulative stress due to their traumatic experiences in residential schools
Y Iwasaki, J Bartlett and J O’neil [59]	*N* = 26	First Nations, Métis	Western Canada Urban	26–69 years M = 9, F = 17	Personal	Mental health/emotional well-being	Some Indigenous individuals with diabetes described cumulative stress due to their traumatic experiences in residential schools
K Jacklin [43]	*N* = 350	First Nations	ON On-reserve	18+ years 143 females interviewed for every 100 males	Personal Familial	General health	Least healthy, most unhappy and the most economically disadvantaged villages had a closer historical relationship to colonial influences (e.g., church, residential school and Indian Agents)
R Jackson, R Cain and T Prentice [60]	*N* = 72	First Nations, Inuit, Métis, Other	ON, BC, AB, MB, Atlantic region	26–54 years *M* = 45, *F* = 23, Transgender = 4	N/A	Mental health/emotional well-being	Some participants attributed experiences of depression to historical trauma and legacy of residential school
LE Jones [61]	*N* = 31,630	First Nations	Canada-wide On/off-reserve Rural/urban/ Northern	49+ years M/F	Personal	Mental health/emotional well-being	Exposure to residential schools led to an increase in smoking and drinking and potentially worse mental health outcomes (e.g., acculturative stress leading to risk health behaviours)
SA Juutilainen, R Miller, L Heikkilä and A Rautio [62]	*N* = 45	First Nations, Sami	ON, Canada; Finland On-reserve	18–80 years M = 18, F = 27	Personal Familial	Mental health/emotional well-being	First Nations participants stated that personal and/or familial attendance at residential school had a negative impact on their health (e.g., language and cultural loss, fractured identity, and negative self-worth resulting in feelings of anger, stress, depression, and low-self-esteem)
V Kaspar [27]	*N* = 13,881	First Nations, Inuit, Métis, Other/multiple identity	Canada-wide Off-reserve Rural/urban/ Northern	34+ years M = 6246, F = 7635	Personal	General health	Residential school attendance predicted negative health status both directly and indirectly through socioeconomic and community risk factors
MJ Kral [63]	*N* = 27	Inuit	NU Northern	17–61 years M = 16, F = 11	Familial	Mental health/emotional well-being	Romantic, family, and intergenerational relations described with suicidality in the context of colonial change. Negative effect of the colonial wound appears to have been on family relations, a serious form of cultural discontinuity
MB Kumar [64]	*N* = 10,306	First Nations, Inuit, Métis	Canada-wide Off-reserve Rural/urban Northern	26–59 years M/F	Personal Familial	Mental health/emotional well-being	First Nations women, Métis men, and Métis women with personal or familial residential school history more likely than those without history to have had suicidal thoughts
MB Kumar and A Nahwegahbow [65]	*N* = 4686 (APS) *N* = 3020 (CCHS–MH)	First Nations, Inuit, Métis	Canada-wide Off-reserve Rural/urban/ Northern	18–25 years	Personal Familial	Mental health/emotional well-being	Personal or familial residential school experience was marginally associated with suicidal thoughts among off-reserve First Nations young adults.
MB Kumar, M Walls, T Janz, P Hutchinson, T Turner and C Graham [66]	*N* = 11,362	Métis	QC, ON, SK, AB, NU Off-reserve Rural/urban/ Northern	20–59 years M/F	N/A	Mental health/emotional well-being	History of residential school experience not significantly associated with suicidal ideation
M Lemstra, M Rogers, A Thompson, J Moraros and R Buckingham [67]	*N* = 603	N/A	SK	N/A	N/A	Mental health/emotional well-being	Attending a residential school was independently associated with depressive symptomatology
M Lemstra, M Rogers, A Thompson, J Moraros and R Buckingham [68]	*N* = 603	First Nations, Inuit, Métis	SK Off-reserve Urban	18–69 years M = 277, F = 253	Personal Familial	Mental health/emotional well-being	Comparing to non-Indigenous IDUs, study found that Indigenous IDUs were more likely to be female and younger, less likely to receive paid income and were more likely to have attended residential school or had a parent/grandparent attend
A Moniruzzaman, ME Pearce, SH Patel, N Chavoshi, M Teegee, W Adam, WM Christian, E Henderson, KJ Craib and MT Schechter [69]	*N* = 605	First Nations, Inuit, Métis	BC Off-reserve Urban	14–30 years M = 313, F = 292	Familial	Mental health/emotional well-being	Having at least one parent who attended residential school was marginally significant with attempted suicide
N Mota, B Elias, B Tefft, M Medved, G Munro and J Sareen [70]	*N* = 1125	First Nations	MB On-reserve	12–17 years M = 520, F = 605	Familial	Mental health/emotional well-being	Suicidality not significantly related to parent/grandparent attending residential school
RT Oster, A Grier, R Lightning, MJ Mayan and EL Toth [71]	*N* = 10	First Nations	AB On-reserve	20+ years M = 7, F = 3	Personal Familial	Mental health/emotional well-being Physical health	Diabetes, broken communities, loss of parenting skills, addictions, suicides, and marital breakups, apprehended children, lifeline (culture) severed, shame, loss of a voice, mental health problems, contaminated families, disarray and chaos, and pain
EA Owen-Williams [72]	*N* = 6	First Nations	BC On/off-reserve Rural	N/A	Personal	Mental health/emotional well-being	Three Elders personally attended residential school and the trauma of these schools was woven throughout each of the interviews. A legacy of resulting anger and alcohol and drug use occurred within communities.
J Reading and B Elias [4]	*N* = 2663	First Nations, Inuit	Canada-wide On-reserve	45+ years M, F	Personal	General health	65% of residential school attendees reported fair or poor health status
LH Robertson [73]	*N* = 3	Aboriginal	N/A	N/A	Personal	Mental health/emotional well-being	Individuals exhibited a cluster of symptoms consistent with Brasfield’s typology, Residential School Syndrome, a specific form of PTSD
A Ross, J Dion, M Cantinotti, D Collin-Vézina and L Paquette [74]	*N* = 358	Indigenous	QC On/off-reserve Rural/urban	18+ years M = 164, F = 194	Personal	Mental health/emotional well-being General health	Residential school attendance was linked to alcohol problems and 83 participants reported that residential school had a negative impact on their health and well-being
C Rotenberg [75]	*N* = 8801	First Nations	Atlantic, QC, ON, Prairies, BC, Territories Off-reserve Rural/urban/ Northern	15+ years M/F	Personal Familial	General health	The study did not detect any significant differences with respect to selected health outcomes analyzed
JP Rothe, P Makokis, L Steinhauer, W Aguiar, L Makokis and G Brertton [76]	*N* = 15	First Nations	AB On-reserve	18–29 years M, F	Familial	Mental health/emotional well-being	Impaired driving, alcohol abuse, and intergenerational impacts due to local people’s traumatic experience with federal government residential schools
D Smith, C Varcoe and N Edwards [77]	*N* = 73	Aboriginal	Location N/A Rural/urban	Age N/A M = 7, F = 66	Personal Familial	Mental health/emotional well-being	Participants described intergenerational effects of residential school as the root of addiction, violence, and poverty among individuals, families, and communities
I Sochting, R Corrado, IM Cohen, RG Ley and C Brasfield [78]	*N* = 127	First Nations	BC	17–81 years M = 89, F = 38	Personal	Mental health/emotional well-being Physical health	Risk factors for PTSD and mental health problems. Somatic complaints, such as chronic headaches, heart problems, high blood pressure, and arthritis
CD Stirbys [79]	*N* = 29	First Nations, Inuit, and Métis	ON	Age N/A F	Personal Familial	Mental health/emotional well-being	Residential schools created initial stressors for those who attended them; the longer-term effects of the children’s experiences showed up in the form of for example, alcoholism, drug abuse, or other self-destructive behaviours
R Stout [40]	*N* = 17	First Nations, Métis, Non-Status, Aboriginal, Undisclosed identity	MB, SK Off-reserve Urban	18–51 years F = 17	Familial	Mental health/emotional well-being	Twelve of the women agreed that familial attendance at residential schools have had an enduring impact on their lives and mental health
R Stout and S Peters [24]	*N* = 6	First Nations	MB	Age N/A F = 6	Familial	Mental health/emotional well-being	Women related how they had a variety of mental health illnesses including depression, eating disorders, workaholism, obsessive-compulsive disorders, self-hate, and low self-esteem
M van Niekerk and A Bombay [80]	*N* = 4934	First Nations	Canada-wide (excl. NU) On-reserve	N/A	Familial	Mental health/emotional well-being	Having a parent who attended residential school put First Nations adults diagnosed with cancer at greater risk for psychological distress compared to those without this family history.
C Varcoe and S Dick [36]	*N* = 30	First Nations, Mixed identity (4 identified as Aboriginal)	BC On/off-reserve Rural	16–58 years F = 30	Personal Familial	Physical health Mental health/emotional well-being	Women’s experiences demonstrated how gender, rural living, poverty, racism, and colonialism intersect and increase risk for health problems, including STIs and HIV
ML Walls, D Hautala and J Hurley [81]	N/A	First Nations	Central Canada, USA On-reserve	Age N/A M/F	Personal Familial	Mental health/emotional well-being	Suicidal behaviour was described by community members as a problem with deep historical and contemporary structural roots
ML Walls and LB Whitbeck [38]	*N* = 853	First Nations; American Indian	Canada-wide, USA On-reserve	Mean age = 39.3 M, F (~72%)	Personal	Mental health/emotional well-being	Bivariate results show that culturally relevant early lifetime (residential school) and adulthood (perceived historical loss) stressors are negatively associated with mental health among adults
D Wardman and D Quantz [82]	*N* = 15	Aboriginal	AB, BC Rural/urban	20–60 years M = 2, F = 13	N/A	Mental health/emotional well-being	Participants related their binge drinking to a broader perception of shame and cultural loss, for some this began in residential schools
K Wilson, MW Rosenberg and S Abonyi [26]	*N* = 51,080	First Nations, Inuit, Métis	Canada-wide On/off-reserve Rural/urban/ Northern	18+ years M, F	Personal	General health	Residential school attendees reported worse health status than the population who did not attend residential school

#### Indigenous identity group

The majority of studies, 43, included First Nations. Eighteen studies involved Inuit and 17 included Métis. In 11, the population was identified as “Aboriginal” or “Indigenous” and did not distinguish between First Nations, Inuit, or Métis. Three studies also included “Other” Indigenous populations that were not further defined, two included multiple identities, one undisclosed identity, and two included non-Canadian Indigenous populations (Sami, American Indian).

#### Geographic location

A total of 14 studies were conducted using national level Canadian data. Seven studies focused on Atlantic Canada; two were conducted in Newfoundland, one in Nova Scotia, one in New Brunswick, and two in the Atlantic region. Six studies were conducted in Quebec, ten studies took place in Ontario, and one in Central Canada. In Western Canada, eight studies took place in Manitoba, eight in Saskatchewan, ten in Alberta, 13 in British Columbia, one in the prairies, and three in Western Canada. Additionally, a few studies were conducted in the territories, with two taking place in the Northwest Territories, and six in Nunavut. Two studies did not specify a geographic location and two were conducted in the USA.

Twenty-four studies considered Indigenous peoples living on-reserve, while 23 involved those living off-reserve. Study participants living off-reserve can be further categorized as living in rural or remote areas, northern communities, or urban areas. Seventeen studies indicated that their participants were from a rural or remote location, 14 included participants in northern communities, and 24 focused on urban populations.

#### Age-sex/gender

Both males and females were represented in the research with 48 studies including both men and women. Five studies included only women, and one solely looked at males. Also, one study included participants who are transgender, one study indicated “other”, and three did not provide a description of the participants’ sex or gender. Regarding age, 46 studies included individuals over the age of 18, whereas 15 included children and youth under the age of 18. Nine studies did not include information on the age of participants.

#### Residential school attendance

In terms of residential school attendance, 42 of the studies reviewed included residential school attendees themselves (personal attendance) and 38 examined the effects of having a parent or other family members who had attended (familial attendance). Four studies did not indicate who had attended residential school.

#### Health outcomes

*General health*: It is evident from the results of this review that personal or familial (e.g. parental or grandparental) residential school attendance is related to health in a multitude of ways. Twelve papers used self-reported health or general quality of life as an outcome measure and found that people who had attended residential schools generally felt as though their health or quality of life had been negatively impacted. Using Statistics Canada’s 2001 Aboriginal Peoples Survey (APS), Wilson and colleagues found that those who had attended residential schools had poorer overall self-rated health than those who did not attend [26], a finding that was reproduced with the 2006 APS by Kaspar [27], who found that 12% of those who had attended residential school reported poor health, compared with 7% of those who did not attend. While this may be attributed to other factors such as aging within the population, the role of residential schools cannot be dismissed [26]. Hackett et al. found that familial attendance at residential school was associated with lower likelihood of reporting excellent perceived health, even after controlling for covariates such as health behaviours, issues with food security and/or housing [28] However, while the studies reveal negative effects in relation to the residential school system, this cannot be said for everyone who attended. For example, some studies have found better overall reported health among those with family members who attended (see, e.g. Feir [29]). *Physical health*: Physical health problems, namely chronic health conditions and infectious diseases, were also apparent in the literature. Thirteen papers related specific physical health conditions to residential school attendance. These included conditions such as HIV/AIDS, chronic conditions (e.g. diabetes, obesity), tuberculosis (TB), Hepatitis C virus (HCV), chronic headaches, arthritis, allergies, and sexually transmitted infections (STIs). In a study by Ghosh [30], participants stated that their experiences at residential school impacted their diets through the higher consumption of carbohydrates, a factor the authors relate to the higher rates of diabetes among this population today. Howard [31] found similar results and suggested that residential schooling contributed to the urbanization of Indigenous peoples in Canada, which has led to diabetes and other problems. Dyck and colleagues also reported that those who attended residential school had a slightly higher prevalence of diabetes than those who did not, although the finding was not statistically significant [32]. Residential school attendance has also been found to be a positive predictor of obesity among younger Métis boys and girls, but a negative predictor among older girls [33]. In addition to chronic conditions, residential school attendance has been associated with poorer sexual health in general [34, 35], infectious diseases such as HIV/AIDS and STIs [36] and has been identified as an independent risk factor for HCV [37]. Corrado and Cohen found that many First Nations people who had personally attended residential schools reported suffering from physical ailments including, chronic headaches, heart problems, and arthritis [5].

*Mental health and emotional well-being*: Mental health, and particularly emotional well-being, was the area of health most commonly identified as affected by residential school attendance. Forty-three studies reviewed found that personal or intergenerational residential school attendance was related to mental health issues such as mental distress, depression, addictive behaviours and substance misuse, stress, and suicidal behaviours. For example, Walls and Whitbeck [38] noted that early lifetime stressors such as residential school attendance are negatively associated with mental health among adults. Corrado and Cohen [5] found that among 127 residential school Survivors, all but two suffered from mental health issues such as PTSD, substance abuse disorder, major depression, and dysthymic disorder. These authors suggest that residential school leads to a specific combination of effects a—“Residential School Syndrome”. Anderson [39] found that residential school attendance among Inuit men was related to mental distress. Familial residential school attendance has been associated with lower self-perceived mental health and a higher risk of distress and suicidal behaviours [28]. Intergenerational effects were found by Stout [40] among women who had parents or grandparents attend residential schools, with women reporting that familial attendance at residential school had had an enduring impact on their lives and mental health.

Substance abuse and addictive behaviours have also been identified as common among those impacted by residential schools. In a study conducted by Varcoe and Dick [36], a participant associates her drinking and drug use to the sexual, physical, emotional, and mental abuse experienced at residential school. Similarly, co-researchers (research participants) in two studies explained their addiction to drugs and alcohol as a “coping mechanism” [44, 54].

Suicide and suicidal thoughts and attempts were associated with personal and familial residential school attendance in several papers. Elias and colleagues [41] found that residential school attendees who suffered abuse were more likely to have a history of suicide attempts or thoughts. Furthermore, non-attendees who had a history of abuse were more likely to report having familial residential school attendance, suggesting that residential schooling might be important in the perpetuation of a cycle of victimization. Youth (12–17 years) participating in the on-reserve First Nations Regional Health Survey who had at least one parent who attended residential school reported increased suicidal thoughts compared to those without a parent that attended [42].

## Discussion

This review aimed to summarize the current literature on residential schools and Indigenous health and well-being using Arksey and O’Malley’s scoping review framework [19]. In general, the empirical literature further documented the wide ranging negative effects of residential schools that had previously been identified by Survivors themselves [15] and confirmed that residential schooling is likely an important contributor to the current health conditions of Indigenous populations in Canada. The studies included revealed a range of poorer physical, mental and emotional, and general health outcomes in both residential school attendees and their families compared with those without these experiences. This included evidence of poorer general health, higher risk of chronic conditions such as diabetes, as well as infectious diseases such as STIs. Many of the studies related residential schooling to poorer mental health, including depressions and substance misuse. Although the majority of studies focused on First Nations, various effects were observed among Métis and Inuit as well, and in urban, rural and reserve populations, and in all regions, strongly suggesting that the effects of residential schooling are felt by Indigenous peoples across Canada. The regional and historical variations in the implementation of residential schooling [10] would lead us to expect geographic variability in these effects. While only one study reviewed examined these differences, it is indicated that variation in health status among community members may be related to various colonial histories in different areas [43]. Importantly, given the vast consequences and predominately negative impact of attendance at these schools, the literature reviewed suggests that younger generations continue to experience the negative health consequences associated with residential schooling. Some of the papers were able to identify specific intergenerational effects, including higher risk of negative outcomes for those whose parents or grandparents attended, whether they themselves were residential school Survivors [9]. Others only considered whether family members had attended, suggesting that the effects are clustered within families, rather than isolating the intergenerational transmission of trauma related to residential schooling.

Overall, the newness of the literature indicates that this is a recent and growing area of research. One of the likely consequences of this is that much of the research reviewed was correlational, and few studies explicitly examined the mechanisms that connected residential school experience to health outcomes. Although some of the studies examining mental health identified substance use resulting from a need to cope with psychological pain [44, 45, 54] or to provide individuals with feelings of regaining power and control [45], most of the studies of physical health effects or general health did not attempt to unpack the range of proximate and mediating factors in the causal chain between residential schooling and the health of Survivors or of their family members.

A strength of this review is that it was conducted systematically and provides methodological accounts to ensure the transparency of the findings. Additionally, the findings of this research highlight the extent and range of the available literature on this important topic in health and suggest areas that require further research. It is important to acknowledge its limitations, however. Firstly, while a scoping review provides a rapid summary of a range of literature, it does not include an appraisal of the quality of the studies included nor provide a synthesis of the data. Secondly, the inclusion of studies is determined by the reviewer’s interpretation of the literature and therefore may be more subjective in nature.

### Implications

The lasting effects of residential schooling on the current Indigenous population are complicated and stretch through time and across generations. It is clear, though, that our understanding of the factors that affect Indigenous peoples’ health should include both the effects of “early, colonization-specific” experiences [27] as well as the more proximate factors, including socioeconomic disadvantages and community conditions [27]. Although this complexity and the impact of colonial policies and practices, such as residential schooling, on other determinants, such as income, education, and housing has been noted [8], there is a need to establish a more comprehensive understanding of the implications of this historical trauma, and particularly of the mechanisms by which intergenerational trauma continues to affect Indigenous peoples’ well-being, including the enduring effects across generations [46].

This would include more research that examines how the effects of residential schooling are mediated or moderated by other social and cultural determinants. For example, the use of ecological frameworks would help researchers and health professionals gain a deeper understanding of how the various levels of context in which the high rates of diseases such as obesity and diabetes have developed have themselves been shaped by colonial policies and by residential schooling in particular. Although isolating the effects of residential schooling on health is important, future empirical analysis should also examine the possible cumulative effects of stressors and traumas, and how these might contribute to the continuing difference between Indigenous and non-Indigenous peoples’ health status [46].

## Conclusions

The findings from this scoping review highlight the importance of considering government policies and historical context as critical to understanding the contemporary health and well-being of Indigenous peoples. As Kirmayer, Tait and Simpson [47] note, this includes other colonial policies, forms of cultural oppression, loss of autonomy, and disruption of traditional life, as well as residential schooling. Better knowledge of how the effects of these historically traumatic events continue to affect communities and individuals may help inform both population health interventions and the care and treatment of individuals. Moreover, identifying the characteristics and conditions of those individuals and communities who have been resilient to the effects of residential schooling may contribute to promoting appropriate supports to limit the transmission of these effects.

## Abbreviations

HCV: Hepatitis C virus
IDU: Injection drug user
PTSD: Post traumatic stress disorder
STIs: Sexually transmitted infections
TB: Tuberculosis

## Footnotes

1 In Canada, “Reserves” are parcels of Crown land set aside for use by particular First Nations communities.
